# Inhibition of Essential Oils on Growth of *Aspergillus flavus* and Aflatoxin B1 Production in Broth and Poultry Feed

**DOI:** 10.3390/toxins14100655

**Published:** 2022-09-22

**Authors:** Bing Han, Guang-Wu Fu, Jin-Quan Wang

**Affiliations:** 1 Key Laboratory of Feed Biotechnology, Ministry of Agriculture and Rural Affairs, Institute of Feed Research, Chinese Academy of Agricultural Sciences, No. 12 Zhong Guan Cun South Street, Haidian District, Beijing 100081, China; 2 China Animal Husbandry Industry Corporation, Ltd., No. 156 Beiqing Road, Haidian District, Beijing 100095, China

**Keywords:** aflatoxin B1, cinnamaldehyde, citral, inhibition, synergy

## Abstract

Aflatoxin B1 (AFB1), a common contaminant in food and feed during storage, does great harm to human and animal health. Five essential oils (thymol, carvacrol, cinnamaldehyde, eugenol, and citral) were tested for their inhibition effect against *Aspergillus flavus* (*A. flavus*) in broth and feed. Cinnamaldehyde and citral were proven to be most effective against *A. flavus* compared to others and have a synergistic effect when used simultaneously. The broth supplemented with cinnamaldehyde and citral was inoculated with *A. flavus* (10^6^ CFU/mL) by using the checkerboard method, and mold counts and AFB1 production were tested on days 0, 1, 3, and 5. Similarly, 100 g poultry feed supplemented with the mixture of cinnamaldehyde and citral at the ratio 1:1 was also inoculated with *A. flavus*, and the same parameters were tested on days 0, 7, 14, and 21. In poultry feed, cinnamaldehyde and citral significantly reduced mold counts and AFB1 concentrations (*p* < 0.05). Results showed that cinnamaldehyde and citral have a positive synergy effect and could both inhibit at least 90% the fungal growth and aflatoxin B1 production at 40 μg/mL in broth and poultry feed, and could be an alternative to control aflatoxin contamination in food and feed in future.

## 1. Introduction

Aflatoxins (AF) constitute secondary metabolites produced by *Aspergillus flavus* and *Aspergillus parasitic* which contaminate a variety of feed ingredients, including peanuts, corn, and cottonseed [1]. Aflatoxin B1 (AFB1) is one of the most toxic members of the aflatoxin family [2]. Previous studies showed that young chicks were especially vulnerable to AF, which may depress feed conversion efficiency and body weight gain, and ultimately cause significant economic losses [3,4]. Because of the carcinogenicity of AF, AF residues in chicken may pose risks to human health [5,6]. AF severely influences the health and growth performance of animals. Therefore, it is necessary to develop a method by which to control the production of AF in feed. Essential oils derived from flavorants often have different capacities to inhibit the growth and toxicity of *A. flavus.*

Essential oils (EO) are complex mixtures of secondary plant metabolites. Moreover, because of their antimicrobial effects, essential oils have been widely used as food preservatives. Over the past several decades, some studies have discovered that various essential oils could also resist fungal growth [7,8]. Kumar [9] reported that the eugenol could inhibit the growth of *A. flavus*. Cinnamaldehyde, thymol, carvacrol et al. also have a strong inhibition to the growth of *A. flavus* [10,11,12]. Therefore, for essential oils derived from plants, it is a safe substitute for antibiotics. The objective of this study was to determine the inhibition of five different essential oils to *A. flavus* and the inhibition of AFB1 production in broth and poultry feed; in addition, a low concentration of essential oils was studied, which was not referred to in the previous studies. Low concentrations of essential oil could not only reduce the harm to animals but reduce the cost of feed industry.

## 2. Results

### 2.1. The Inhibition of Different Concentration of Essential Oils against A. flavus

**Figure 1:**
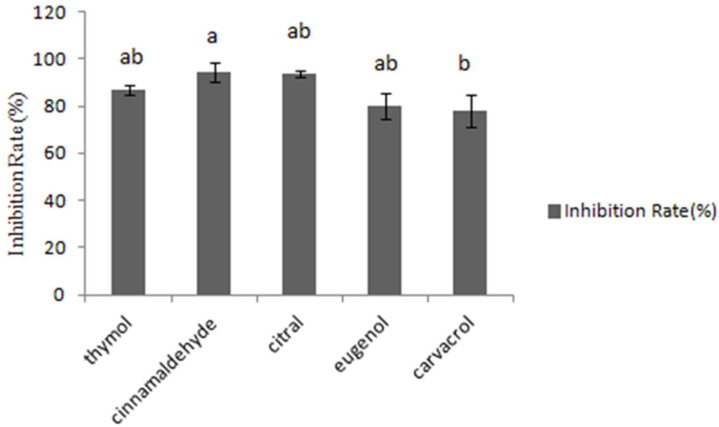
Inhibition of different essential oils at the concentration of 40 μg/mL. Different small letters in the same row (a, b) denote a significant difference *(p* < 0.05) among values, based on Tukey’s test.

The fungal growth inhibition was assessed by testing absorbance at 600 nm. In Figure 1, cinnamaldehyde inhibited 94.4% of the four fungal growth types, while other tested essential oils inhibited 93.7%, 86.9%, 80.1%, 78%, respectively. Consequently, it was concluded that cinnamaldehyde and citral had the most significant inhibitory effects on the growth of *A. flavus* CGMCC 3.2890 at the concentration of 40 μg/mL, and inhibition rate of cinnamaldehyde was higher compared to carvacrol (*p* < 0.05) but no significance compared to other essential oils (*p* > 0.05).

Consequently, cinnamaldehyde and citral were screened for further research with regard to their inhibitory effects on the growth of *A. flavus* in feed. Representative strain *A. flavus* CGMCC 3.2890 was considered for further research.

### 2.2. MIC Tests and Synergy Effects of the Best Effect of Essential Oils on Fungal Growth in Broth by Using Checkerboard

The synergy effect of cinnamaldehyde and citral against *A. flavus* CGMCC 3.2890 was tested by using the checkerboard method. The modal minimum inhibition concentration (MIC) results are presented in Table 1. The two essential oils synergistically affected *A. flavus* CGMCC 3.2890 as shown in Table 2.

From the results of Table 2 and the FIC value (FIC = 0.5), it could be concluded that cinnamaldehyde and citral had the synergy effects when used simultaneously.

### 2.3. Effects of Essential Oils on Fungal Growth and AFB1 Production in Poultry Feed

**Figure 2:**
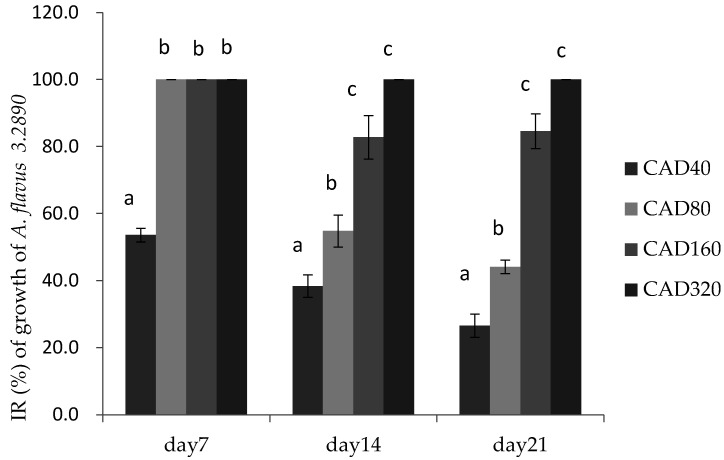
Inhibition rate (IR) of cinnamaldehyde and citral on the growth of *A. flavus* 3.2890 in feed at 40 μg/mL (CAD40), 80 μg/mL (CAD80), 160 μg/mL (CAD160), and 320 μg/mL (CAD320). Different small letters in the same row (a, b, c) denote a significant difference (*p* < 0.05) among values, based on Tukey’s test.

**Figure 3:**
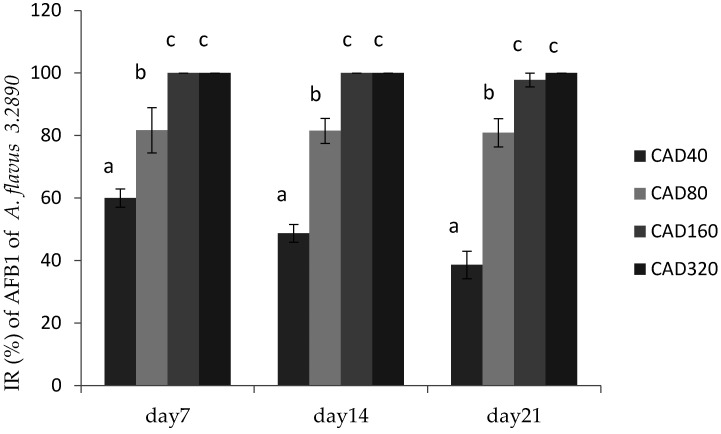
Inhibition rate (IR) of cinnamaldehyde on AFB1 production by *A. flavus* CGMCC 3.2890 in feed at 40 μg/mL (CAD40), 80 μg/mL (CAD80), and 160 μg/mL (CAD160). Different small letters in the same row (a, b, c) denote a significant difference (*p* < 0.05) among values based on Tukey’s test.

Cinnamaldehyde and citral were screened for further research with regard to their inhibitory effects on the growth of *A. flavus* in poultry feed on days 0, 7, 14, and 21, respectively. Representative strain *A. flavus* CGMCC 3.2890 was considered for further research in feed. The effects of combination of two essential oils on the growth of *A. flavus* CGMCC 3.2890 in feed were shown in Figure 2 and Figure 3, respectively.

Figure 2 showed that on day 7 there was no significance among the CAD80, CAD160, and CAD320 (*p* > 0.05) with IR almost 100%, but on days 14 and 21, the growth of molds was significantly decreased at 160 μg/mL (CAD160) and 320 μg/mL (CAD320) compared to CAD40 treatment, respectively, at the end of the storage period. Therefore, the addition of cinnamaldehyde and citral could suppress the molds sprouting in feed during the first week. 

Figure 3 showed the inhibitory effect of cinnamaldehyde and citral on AFB1 production by *A. flavus* 3.2890 in feed, where it could be seen that CAD160 and CAD320 treatments could completely inhibit the productions of AFB1 on day 14 and day 21 (*p* < 0.05) compared to other groups, and CAD40 and CAD80 could reduce the production of AFB1 to some extent (*p* < 0.05). However, with the time going, a high concentration could still totally inhibit fungus growth and AFB1 production on day 21, while the IR of low centration decreased, but compared to CT treatment, the low concentration could still play an important inhibition role to some extent.

## 3. Discussion

In this research, a high-concentration treatment could totally inhibit the growth of fungus and AFB1 production even on day 21, perhaps because the essential oils have killed the fungus, and the low concentration of essential oils probably just inhibited the fungus. In line with Mahnoud [13] and López–Malo et al. [14], the results of the present study showed that the growth of *A. flavus* CGMCC 3.2890 was inhibited by the five EOs, which was in accordance with Kumar et al. [9]. However, in the former studies, the concentrations of inhibition were all high, rarely studying the inhibition capability of low concentrations. Our research proved that low concentration also has inhibition ability against *A. flavus.* Our results showed that the cinnamaldehyde and citral suppressed the growth of *A. flavus* at low concentrations. Sun et al. [15] also reported that the germination of *A. flavus* was delayed by cinnamaldehyde in PDA medium when administered at 79.29 mg/L, whereas our concentration was only 40 μg/mL. The reason why cinnamaldehyde had the best inhibition ability was perhaps related to its special structure, such as aldehyde and phenol, which could attack the cell membrane or cell wall. Nogueira et al. [16] indicated that essential oil of *Ageratum conyzoides* changed the ultra-structure of *A. flavus*, which was more evident in the endomembrane system, such as mitochondria, thus inhibiting the growth of *A. flavus.* Sun et al. [15] reported that the diameter of the spore size linearly decreased with the increase of concentration of essential oils. The probable mechanism of cinnamaldehyde and citral needs further study.

The checkerboard method is a common method by which to evaluate the synergy effect of different drugs. The results showed that cinnamaldehyde and citral could synergistically affect *A. flavus*, which was rarely reported before.

Cinnamaldehyde and citral dose-dependently inhibited AFB1 production in the liquid medium, which was in accordance with our results in the feed. The mechanism of decreased AFB1 production by cinnamaldehyde and citral may be related to the downregulation of the expression of key genes for AFB biosynthesis, such as *aflC*, *nor1* and *norA* [16].

In the feed industry, essential oil is usually used as an odorant to increase feed intake, and acidifier product is often used to inhibit fungus, but the dosage of acidifier in feed is high, which may cause the negative effect on the performance of animals. Consequently, essential oil could be a good replacement for acidifier. Essential oils could not only inhibit the fungus, but have many other positive effects on animal production. Thymol was proven to increase the polyunsaturated fatty acid in egg yolk [17], perhaps for its antibacterial and antioxidant properties, which was also proven in other studies [18]. Moreover, essential oils could be improved by increasing the oleic acid content [19], and thiobarbituric acid reactive substances (TBARS) values could be lowered and the color parameters could be increased during storage when using essential oils [20]. Now only rosemary extract and oregano essential oils are permitted to be used as feed additives, but in the future, more essential oils would probably be applied as feed additives.

## 4. Conclusions

AFB1 do great harm to the health of human and animals. The research showed cinnamaldehyde and citral could be an alternative to control aflatoxin contamination in food and feed in future.

## 5. Material and Methods

### 5.1. Microbial Cultures

The following microbial strain were selected for their relevance in the feed industry: *A. flavus* CGMCC 3.2890 was obtained from China General Microbiological Culture Collection Center (CGMCC). The fungi strain was subcultured in potato dextrose agar (PDA) at 28 °C for five days. A spore suspension (approx. 10^6^ CFU/mL) was prepared with potato dextrose broth (PDB).

### 5.2. Preparation of Essential Oils

Thymol (Sinopharm Chemical Reagent Co., Ltd., Beijing, China, ≥99.0%), cinnamaldehyde (Sinopharm Chemical Reagent Co., Ltd., Beijing, China, ≥99.0%), citral (Sinopharm Chemical Reagent Co., Ltd., Beijing, China, ≥97.0%), eugenol (Sinopharm Chemical Reagent Co., Ltd., Beijing, China, ≥98.5%), and carvacrol (J & KCHEMICA, Beijing, China, ≥98.0%) were mixed with potato dextrose broth (PDB) containing ethanol (5%; *v*/*v*) and tween 80 (0.5%; *v*/*v*) at 1000 μg/mL, 200 μg/mL, and 40 μg/mL, respectively. PDB solution was prepared according to the above method (devoid of essential oils) served as the control.

### 5.3. Screening of Best Effect of Essential Oils on Mold Growth in Broth

The modified micro-plate assay used in this study has already been described in detail by Gorran et al. [21]. Briefly, EOs at different concentrations (0, 40, 200 and 1000 μg/mL) were screened for inhibiting *A. flavus* growth. In 96-well micro-plates (Costar^®^, 3599, Corning, NY, USA), 160 μL PDB and 20 μL of different concentrations of EOs were mixed with 20 μL of four different strains of prepared *A. flavus* spores (at the concentration of 10^6^ CFU per well), and shaken overnight at 28 °C. The fungal growth was determined by measuring the absorbance at 600 nm of fungal culture in 96-well micro-plates by using a micro-plate reader (model 680, BIO-RAD Laboratories, Inc., Hercules, CA, USA) for 24 h and 48 h [22]. All assays were performed in triplicates. The essential oils of best inhibition effect were chosen for the next trials.

### 5.4. MIC Tests and Synergy Effects of the Best Effect Essential Oils on Fungal Growth in Broth

The MICs of two essential oils’ best inhibition effects were separately determined by broth microdilution method by using 96 kits. The MICs were tested in replicates of six. The MIC was defined as the lowest concentration of completely inhibiting the growth of *A. flavus*. The inoculums were approximately 1 × 10^6^ CFU/mL in each well.

The synergy effects of the two essentials were determined by using the checkerboard method. The concentration range of each essential oil in combination ranged from 1/4 MIC to 2 MIC. Dilutions of two essential oils were made with a twofold diluter [14]. The initial inoculum was approximately 1 × 10^6^ CFU/mL. The fungal growth was determined by measuring the absorbance at 600 nm of fungal culture in 96-well micro-plates by using a micro-plate reader (model 680, BIO-RAD Laboratories, Inc., Hercules, CA, USA) for 24 h.

To evaluate the effects of combinations, the fractional inhibition concentration (FIC) was calculated for each essential oil in each combination [22]. The following formulas were used to calculate the FIC index. The results were expressed as four situations, including synergy (FIC ≤ 0.5), additive (0.5 < FIC ≤ 1), indifference (1 < FIC ≤ 4) or antagonism (FIC > 4). A and B separately stands for the two tested essential oils:

FIC = (MIC_A + B_)/(MIC_A_) + (MIC_A + B_)/(MIC_B_). (1)

### 5.5. Effects of the Best Effect Essential Oil on Fungal Growth and AFB1 Production in Poultry Feed

Prior to mixing with cinnamaldehyde, broiler feed free of any toxin binder was sterilized at 121 °C for 20 min followed and the moisture of the feed was adjusted to 17% (on dry basis) with sterile water. Then the feed was inoculated with each mold separately by using the method described by Yin et al. [23], wherein *A. flavus* CGMCC 3.2890 was added to 200-g portions of feed to obtain 5.5 log CFU/g feed, and mixed well. After inoculation, cinnamaldehyde and citral were added at 0, 40, 80, 160, and 320 mg/kg (CT, CAD40, CAD80, CAD160, CAD320) feed totally followed by incubation at 28 °C in 500 mL Erlenmeyer flasks, sealed with rubber closures. A 20-g portion of the feed was sampled on days 0, 7, 14, and 21, of which 10 g was used for mold enumeration and 5 g for AFB1 detection, respectively, to calculate the inhibition rate (IR) of *A. flavus* CGMCC 3.2890 and AFB1. Each treatment was repeated three times.

The inhibition rate (IR) of *A. flavus* CGMCC 3.2890 was calculated according to the following formula:

IR (%) = (ΔOD_c_ − ΔODx)/ΔOD_c_ × 100. (2)

IR means the inhibition rate of *A. flavus*, ΔOD_c_ means the difference value of the OD of CT treatment of day (7, 14, 21) and day0, ΔOD_x_ means the difference value of OD of the treatments (CAD40, CAD80, CAD160, CAD320) on day x (day 7, 14, and 21) and day 0 respectively;

IR of AFB1 was calculated according to the following formula:

IR (%) = (ΔAFB_c_ − ΔAFBx)/ΔAFB_c_ × 100. (3)

IR means the inhibition rate of AFB1, ΔAFB_c_ means the difference value of the concentration of AFB1 of CT treatment on day x (day 7, 14, 21) and day0, ΔAFBx means the difference value of the concentration of AFB1 of of the treatments (CAD40, CAD80, CAD160, CAD320) on day x (day 7, 14, 21) and day 0, respectively.

### 5.6. Determination of Mold Counts

To enumerate *A. flavus* in the control and treated feed, 10 g portions of feed samples were added to 100 mL of PBS in sterile glass flasks, and blended in a shaker for 30 min. The feed homogenate was serially diluted (1:10) in PBS, and 0.1 mL aliquots from appropriate dilutions were surface-plated on duplicate PDA plates, and incubated as previously described.

### 5.7. Determination of AFB1 in Feed 

The concentrations of AFB1 were determined by using a commercial ELISA Kit (HEM 00496, Huaan Magnech Bio-Tech Co., Ltd., Beijing, China). All the procedures were performed on the basis of manufacturer’s instructions and the absorbance was determined by using a micro-plate reader. The AFB1 kit is an indirect competitive enzyme-labeled immunoassay. The AFB1 antigen is pre-coated on the wells. The pre-coated antigen competes with the AFB1 antibody (antibody solution) with AFB1 in the sample, anti-AFB1 antibody binds to the AFB1-HRP enzyme conjugate. The substrate solution was pipetted into the wells to convert the color. The color of unknown samples is compared to the color of the standards and the AFB1 concentrations of the samples were derived.

Samples were prepared by weighing out a 5.0-g comminuted sample into a 100-mL triangular flask with a stopper. A total of 25 mL of 60% methanol solution was added and blended vigorously for 10 min on a vertex. The sample was transferred to a centrifuge tube and centrifuged for 5 min at 4000 r/min. A total of 1.0 mL of the top-layer liquid was transferred to a new tube, and 4.0 mL of deionized water was added and blended for 5 s. A total of 50 μL of the solution was taken for assay.

### 5.8. Statistical Analysis

Data from this study was analyzed with one-way ANOVA followed by Tukey’s multiple range test; data were expressed as the mean ± SE by Tukey’s multiple range test. Data were expressed as significant if *p* was less than 0.05. All statistical analyses were performed by SPSS 25.0 (SPSS Inc., Chicago, IL, USA).

## Figures and Tables

**Table 1:** Modal MIC results by broth microdilution.

Essential Oils	MICs
Cinnamaldehyde	80 ppm
Citral	80 ppm

**Table 2:** Synergy method results.

	Cinnamaldehyde	0	1/4 × MIC	1/2 × MIC	1 × MIC	2 × MIC
Citral						
0		0.439 ± 0.012	0.428 ± 0.022	0.427 ± 0.038	0.377 ± 0.034	0.376 ± 0.027
1/4 × MIC		0.419 ± 0.009	0.401 ± 0.028	0.369 ± 0.029	0.371 ± 0.011	0.371 ± 0.051
1/2 × MIC		0.418 ± 0.025	0.383 ± 0.003	0.425 ± 0.082	0.372 ± 0.028	0.374 ± 0.044
1 × MIC		0.419 ± 0.018	0.392 ± 0.008	0.372 ± 0.022	0.368 ± 0.036	0.375 ± 0.051
2 × MIC		0.406 ± 0.003	0.392 ± 0.053	0.366 ± 0.004	0.377 ± 0.017	0.376 ± 0.032

## Data Availability

Not applicable.
